# Supplementary material for: Tactile sensitivity and motor coordination in infancy: Effect of age, prior surgery, anaesthesia & critical illness
Source: PLoS One. 2022 Dec 30;17(12):e0279705. doi: 10.1371/journal.pone.0279705 (PMC9803162; doi:10.1371/journal.pone.0279705)
Supplement: S1 File — (PDF) [file pone.0279705.s001.pdf]

Tactile sensitivity and motor coordination in infancy: effect of age, prior surgery, anaesthesia and critical illness

Laura Cornelissen, Ellen Underwood, Laurel J Gabard-Durnam, Melissa Soto, Alice Tao, Kimberly Lobo, Takao K Hensch & Charles B Berde

Supporting information

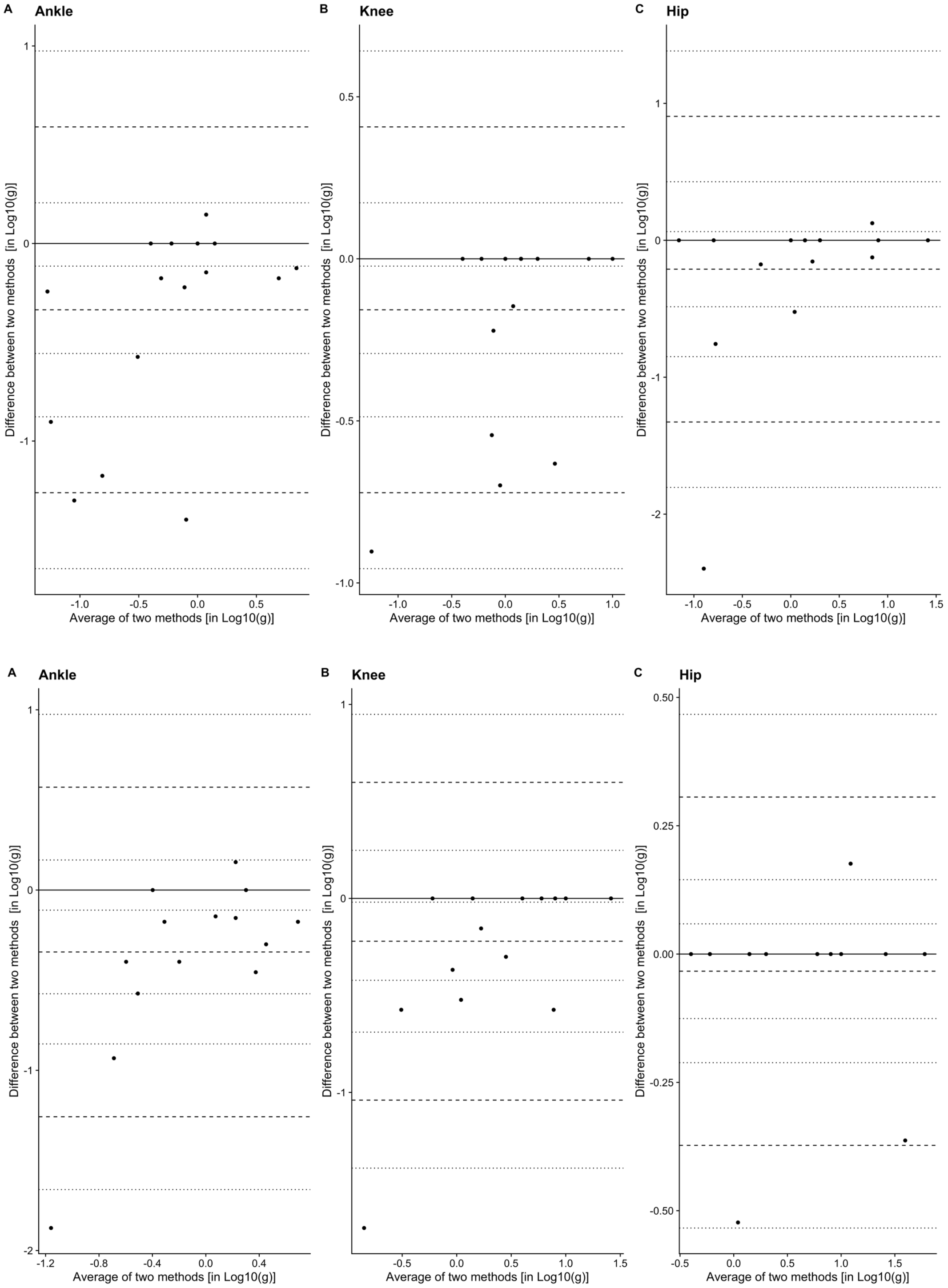

S1 Fig: Bland Altman plots for single vs multiple trials to summarise lower limb flexion threshold at age 1 to 3 months (top row) and age 4 to 7 months (bottom row). Dashed horizonal lines represent the bias and limits of agreement.

		1 to 3m N = 66		4 to 7m N = 49	
**Hair unit**	**Forge-grams**	**Completed**	**Missing** (hair not applied)	**Completed**	**Missing** (hair not applied)
1	0.008	66	0	49	0
2	0.02	66	0	49	0
3	0.04	66	0	49	0
4	0.07	66	0	49	0
5	0.16	66	0	49	0
6	0.4	66	0	49	0
7	0.6	66	0	49	0
8	1.0	66	0	47	2
9	1.4	63	3	47	2
10	2.0	52	14	46	3
11	4.0	40	26	41	8
12	6.0	33	33	37	12
13	8.0	22	44	31	18
14	10.0	17	49	26	23
15	15.0	14	52	19	30
16	26.0	11	55	15	34
17	60.0	8	58	7	42
18	100	2	64	3	46
19	180	2	64	2	47
20	300	1	65	1	48

S1 Table: Frequency table showing von Frey monofilament conversion units and number of studies with missing data i.e. where hair was not applied because nociceptive threshold was already established.

Dependent variable	Predictor	Estimate	95% CI	*t*	*P*
Tactile sensitivity threshold	**Intercept**	**-0.59**	**-0.98 to -0.19**	**-2.94**	**< .001**
(N = 65,	Age at study	0.09	0 to 0.19	1.97	.051
Occasions = 115)	Sex	0.04	-0.22 to 0.31	0.31	.76
	State	-0.14	-0.48 to 0.19	-0.85	.4
Nociceptive threshold	Intercept	0.23	-0.07 to 0.53	1.53	.13
(N = 65,	**Age at study**	**0.14**	**-0.07 to 0.21**	**3.86**	**< .001**
Occasions = 115)	Sex	0.02	-0.19 to 0.22	0.15	.89
	State	-0.08	-0.33 to 0.17	-0.61	.55

S2 Table: Estimated model fixed effects with predictors known to have an effect on sensitivity in the adult literature (state, sex).

Age is a significant predictor for Nociceptive Thresholds, while Tactile Sensitivity Threshold had no clear predictors based on the predictors included in the model for this sample. For tactile sensitivity modelling of random effects from subjects, ICC was low at 0.08, σ^2 was 0.39, $\tau_{00}$was 0.03. For nociceptive threshold modelling of random effects from subjects, ICC was low at 0.09, σ^2 was 0.22, and τ_{00} was 0.02.

Significant predictor ($p < 0.05$) is shown in bold. Abbreviations: CI = Confidence Interval; ICC = Intraclass-Correlation Coefficient; *P* = p value; *t* = t statistic; σ^2 = within-group variance; τ_{00} = between-group-variance.

**Dependent variable**	**Model**	**Predictor**	**Estimate**	**95% CI**	***t***	***P***
Ankle-flexion	Model 1	Intercept	-0.18	-0.41 to 0.06	-1.5	.14
(N = 64,		**Age at study**	**0.07**	**0 to 0.13**	**2.12**	**.036**
Occasions = 110)		Number of surgeries	0.01	-0.05 to 0.06	0.26	.8
	Model 2	Intercept	-0.17	-0.41 to 0.06	-1.47	.15
		**Age at study**	**0.07**	**0.01 to 0.14**	**2.18**	**.031**
		Duration GA	0	-0.01 to 0.01	-0.16	.87
	Model 3	Intercept	-0.18	-0.42 to 0.06	-1.52	.13
		**Age at study**	**0.07**	**0.01 to 0.14**	**2.23**	**.028**
		Duration GA	0	-0.02 to 0.01	-0.55	.58
		Duration ICU stay	0	0 to 0.01	0.63	.53
Knee-flexion	Model 1	Intercept	-0.12	-0.35 to 0.1	-1.07	.29
(N = 64,		**Age at study**	**0.13**	**0.07 to 0.2**	**4.22**	**< .001**
Occasions = 112)		Number of surgeries	0.01	-0.04 to 0.06	0.4	.69
	Model 2	Intercept	-0.12	-0.34 to 0.11	-1.04	0.30
		**Age at study**	**0.13**	**0.07 to 0.2**	**4.28**	**< .001**
		Duration GA	0	-0.01 to 0.01	0.05	.96
	Model 3	Intercept	-0.11	-0.34 to 0.12	-0.98	.329
		**Age at study**	**0.13**	**0.07 to 0.2**	**4.19**	**< .001**
		Duration GA	0	-0.01 to 0.02	0.39	.694
		Duration ICU stay	0	-0.01 to 0	-0.53	.6
Hip-flexion	Model 1	Intercept	-0.09	-0.37 to 0.18	-0.67	.50
(N = 62,		**Age at study**	**0.17**	**0.09 to 0.24**	**4.42**	**< .001**
Occasions = 93)		Number of surgeries	0.02	-0.05 to 0.09	0.59	.56
	Model 2	Intercept	-0.09	-0.37 to 0.18	-0.65	.52
		**Age at study**	**0.17**	**0.09 to 0.24**	**4.48**	**< .001**
		Duration GA	0	-0.01 to 0.02	0.37	.71
	Model 3	Intercept	-0.09	-0.36 to 0.19	-0.64	.53
		**Age at study**	**0.17**	**0.09 to 0.24**	**4.46**	**< .001**
		Duration GA	0	-0.02 to 0.02	-0.06	.95
		Duration ICU stay	0	-0.01 to 0.01	0.51	.61

S3 Table: Estimated model evaluating fixed effect predictors of ankle, knee or hip flexion threshold.

Age is a significant predictor in ankle, knee and hip lower limb flexion thresholds in this sample of infants aged from 1 to 7 months. Random effects from participants for ankle-flexion indicated σ^2 was 0.21, $\tau_{00}$was 0.0; for knee-flexion indicated σ^2 was 0.20, $\tau_{00}$was 0.0; and for hip-flexion indicated σ^2 was 0.19, $\tau_{00}$was 0.08 for Models 1, 2 and 3.

Significant predictor ($p < 0.05$) is shown in bold. Abbreviations: CI = Confidence Interval; ICC = Intraclass-Correlation Coefficient; *P* = p value; *t* = t statistic; σ^2 = within-group variance; τ_{00} = between-group-variance.
