# Supplementary material for: Tactile sensitivity and motor coordination in infancy: Effect of age, prior surgery, anaesthesia & critical illness
Source: PLoS One. 2022 Dec 30;17(12):e0279705. doi: 10.1371/journal.pone.0279705 (PMC9803162; doi:10.1371/journal.pone.0279705)
Supplement: Attachment — Submitted filename: 2022-11-14_02_Response-to-Reviewers.pdf [file pone.0279705.s003.pdf]

REVIEWERS' COMMENTS

DETAILED COMMENTS FROM Reviewer #1:

Thank you for the opportunity to review "Tactile sensitivity and motor coordination in infancy: effect of age, prior surgery, anesthesia and critical illness" This manuscript is well written and interesting and provides useful and clinically relevant. Here, the authors provide increasing force application stimuli to infants' foot and record the motor response. They tested whether multiple testing or a single session were necessary, the effects of hospitalization and general anesthesia, and the effects of infant age. This was an impressively described study. I rarely suggest accepting a manuscript after a first read. I certainly have some comments below, but that it overall, it was clear and worthy of publishing as is.

Introduction. Line 74: Could you add more here about this 'critical need'? Perhaps describing more how the results of these tests are used in research or clinical purposes would help me understand why there is a critical need for better testing. You talk about this in the discussion and clinical implications, just moving it into the intro would help. Very clear specific aims and hypotheses.

Thank you for your suggestions. We agree and have edited the Introduction to provide examples of how sensory testing protocols are used in research or the clinic as follows:

> "There is a critical need to identify brief and feasible sensory testing protocols that can be performed in pre-and non-verbal populations and provide useful comparative data for research and clinical purposes. The results of these quantitative sensory tests have implications for use in research and in the clinic when examining nerve function for pain assessment (refs), disorders associated with atypical sensory processing (refs), in clinical trials of analgesics and local anesthetics (refs), and in safety testing of novel spinal therapeutics for neurodegenerative disorders (refs).

Methods
Line 145: I'm curious about the two positions- sitting vs lying down. In sitting, the flexion movement is against gravity, while lying down, it is across gravity. Does this have any affect on the LE movement?

Infants were positioned on a caregiver's lap so that legs were free to move – when either sitting or lying down over their caregiver's lap. All infants displayed clear nociceptive reflexes, measurable as lower limb withdrawal and flexor muscle activity, in response to large von Frey hair forces. Working with two positions instead of one is a limitation, but these are natural and relaxed positions used when testing in awake cooperative children at these ages and the effect of movement against gravity should be minimal.

Line 147: What 'gross motor activity' was recorded?
"Gross motor activity" was used to indicate to the reader that large movement was recorded i.e. detectable to the visible eye. This movement includes ankle, knee and/or hip flexion. We have edited the text to provide clarity as follows:

> "Gross motor activity was recorded following stimulus application. Gross motor activity consisted of either (i) ankle-flexion (dorsiflexion), (ii) knee-flexion, and (iii) hip-flexion."

Line 149: I'm confused about what you mean by trial. Is one trial equivalent to a series of stimuli until a threshold is reached? It references S1 fig at the end of this paragraph- I like this figure and think it helps clarify the paradigm. Would you consider adding it as a figure rather than supplementary? Is there also a way on this figure to add in the Nociceptive and tactile thresholds?
Thank you. One trial is equivalent to a series of stimuli that are applied until nociceptive threshold is reached. Two to three trials were performed in each subject so that an average threshold could be determined. We have provided clarity in the study protocol by moving S1 Fig to the main text (new Fig 2) and edited the figure to include clarity on thresholds.

Line 167: If participant is lying down, are these definitions different? Are the feet out straight during the test? And if so, how is knee flexion defined, since the thigh would likely also move?

We studied subjects who were in the upright position in their parents lap or who were lying down. Subjects who were classed as lying down were held in their parents lap with their feet dangling. Figure R1 is an example of a 2-month infant who was studied while lying on a caregiver's lap.

Figure R1 is a screenshot taken from a video frame. The experimenter is shown on the left, holding the von Frey hair (at rest). The subject is shown on the right side of the image, in their caregiver's lap. The top part of the screenshot is cut-off to protect the identities of the experimenter and caregiver; a black box is placed over the subject's face per HIPPA guidelines.

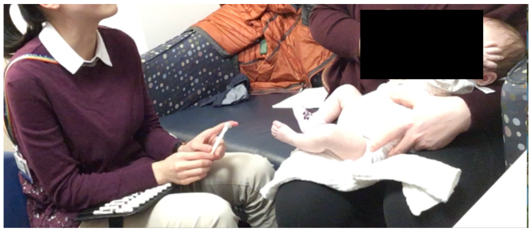

Figure R1: Screenshot of 2-month infant lying on caregiver's lap

Line 180: Could you add an overall significance level here also?
We have edited the Methods to include a statement on the confidence level as follows:

> "P values < 0.05 are considered statistically significant."

Results
Nice tables and figures.
Thank you!

Discussion

Line 480- can you add some citation for this first sentence?
Thank you for the suggestion. Citations are now included as follows:

> "In our study, nociceptive threshold increases with age, consistent with previous studies in premature infants (refs), older children (refs), and young adults (refs).

Line 508- I'm not sure how these last two sentences relate to your findings. They seem to contradict it. Can you expand on this?

Thank you. We originally provided the sentences to acknowledge that other groups have reported changes in cutaneous sensitivity in children exposed to painful and stressful procedures in early life, and to reason that we did not see similar effects due to our study sample being stimulated at sites located away from areas exposed to tissue damage. We have removed the sentences to avoid confusing the reader, and added an extra sentence as follows:

> "Future work warrants testing subjects at body sites that are located closer to areas exposed to tissue damage."

DETAILED COMMENTS FROM REVIEWER #2:

This article aimed to study tactile sensitivity in the infant period and the associated coordination of lower limbs in children of different groups. The study was conducted on two groups of children of different ages. In addition, typically developing children were compared with infants with prior hospital experience such as surgery, anesthesia, or critical conditions. The authors of the article described in detail the movements of the ankle, knee, and hip as responses to mechanical stimuli of varying intensity degrees. The likelihood of ankle, knee, and hip flexion has been shown to increase with greater stimulus intensity.

One of the strong points of the article is that the reliability of single and averaged summary measurements of the threshold of sensitivity was confirmed, which allows us to consider this method as sensory testing for young children. However, it could seem that there is a low degree of objectivity in the proposed assessment, due to the fact that muscle reactions can be visually unobservable, but clearly manifested in the limb's muscle tone, in particular in children who earlier had critical conditions.

We agree with the reviewer's comment. We have acknowledged this limitation in the Discussion and suggested that EMG offers utility as a tool to provide an objective and quantifiable measures of motor activity that might offer increased sensitivity compared to visual observation.

The Limitations section is written as follows:

> " The second limitation relates to the reliance of observational methods to assess the presence of motor activity, rather than surface electromyography as a tool to objectively and quantitatively determine the degree of muscle activity generated. Electromyography has utility in patient populations who are non-verbal with motor issues, and who may be able to perceive a tactile stimulus but may not be able to mount a reflex movement or behaviour in response."

Another point for clarification is that in this article as I understood, premature babies from 32 weeks of gestational age were also included in the group of typically developing healthy children. But in the literature (Einspieler,2016; Engle WA, 2007; Natarajan G, 2016), this age is considered as a significant degree of prematurity. In this regard, I would like to know the possibility of comparing the thresholds of tactile and nociceptive sensitivity in children of two groups - premature and full-term babies.

The reviewer raises an excellent point. We had planned to look at prematurity as a covariate. The final numbers of premature-born children who were enrolled in the study were too low to perform any meaningful statistical testing (n = 3). Figure R2 shows the group averaged sensory threshold data in Control participants grouped by premature or term birth.

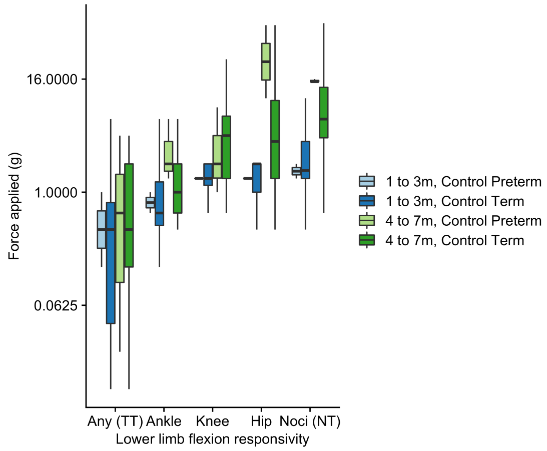

Figure R2. Sensory thresholds for lower limb flexion responsivity in the Control group of children born preterm and term

Minor points.

1. It is possible that there is a mismatch between two values - in Tabl.1, in the second column "Control" I can read that number of participants in this group is 18, but in the line 290 it is written that n =19.

Thank you for spotting this error.

2. In the Tabl.1 In the first column it is written that "age at birth" is indicated only in months, but actually in weeks and in months.

The relevant table units now read "Age at birth, wks"

In general, the above questions do not reduce this study's scientific and practical value. In this regard, I recommend the article for publication.

Thank you for your positive and constructive feedback.

DETAILED COMMENTS FROM REVIEWER #3:

Introduction. Line #88.

Insert references in the sentence "Studies of tactile and nociceptive responses in paediatrics have focused primarily on premature and term infants, or on older children of the age and cognitive development that requires the use of verbal responses, and generally provide data from a single point in time".
We have included references to support the statement as follows:

> "Studies of tactile and nociceptive responses in paediatrics have focused primarily on premature and term infants (refs), or on older children of the age and cognitive development that requires the use of verbal responses (refs), and generally provide data from a single point in time."

Methods
1. It is necessary a more detailed description of the foot region of the stimulation. Was in the center of the plantar surface or in the level of the metatarsus head or in the calcaneous region or any other region?
Thank you. We have edited the Methods text to provide a more detailed description as follows:

> "stimuli at graded weights were applied to the plantar surface of the foot (at the level of the 1st to 3rd metatarsus head) at rest"

2. How many researchers did the stimulation?
Four researchers were responsible for applying the von Frey hairs to the foot. All researchers received training in sensory testing. The advantage of using mechanical von Frey hairs is that that a set force is delivery when the hair is applied to the foot perpendicularly.

3. It is not clear how many raters carried out the motor evaluation. Were there two or more?
The motor evaluation i.e. posthoc video assessment, was performed by two raters (EU, LC).

4. Please insert the confidence level of the statistics.
We have edited the Methods to include a statement on the confidence level as follows:

> "P values < 0.05 are considered statistically significant."

DETAILED COMMENTS FROM REVIEWER #4:

To the best of my knowledge, this is one of the first studies evaluating sensitivity to tactile and nociceptive stimuli in infants. The authors stimulated the foot sole of 74 infants with von Fey filament and reported the tactile/nociceptive threshold defined as the minimum stimulus (in grams) that elicited an ankle/knee/hip join flexion, or a fast leg withdrawal, respectively. The experimental question is novel and interesting, the major limitation of the study is that the methodology was rather simple and therefore it only allow the authors to record basic measurements. For example, the movement of the infants was recorded with iPhone 5S video recording. I assume that the distance of the camera and the angle with respect to the scene was highly variable across participants. Gross motor-response was assessed by observing video with a standard video player. Automatic human pose estimation using deep learning techniques (for example with open pose or deep lab cut) would be a better solution towards an objective assessment. See below for my detailed comments

Major issues

1. The authors uses video recordings (iPhone 5S, Apple inc., CA) to capture the subject's motor response following each stimulus application. In future work, I would recommend to use a camera at a fixed distance to the participant and a software for automatic pose estimation. As discussed in the paragraph "Limitations", electromyography may be also useful to detect a small response in particular in flexor muscles.
Thank you for the recommendations on using software for automatic pose estimation. Its exciting to see how the field of motion analysis is advancing.

2. "Motor activity was inspected frame-by-frame at a 1Hz sampling rate" not clear, 1Hz was the sampling rate for the video assessment or for the recording? If (as I assume) it was the sampling rate for the assessment, which was the sampling rate for the video recording?
Videos were recorded at a 30Hz sampling rate, and video assessment of motor activity was inspected frame-by-frame (1 frame per 30ms). We have added in detail to the Methods-Sensory testing protocol, and Methods-Data analysis sections to make the sampling rate clear to the reader as follows:

> "Video recordings (iPhone 5S, Apple inc., CA) captured the subject's motor response following each stimulus application for post-hoc analysis. The rear-facing camera on the smartphone was used to capture 1080p HD video at 30 frames per second.
> […]
> "Motor activity was inspected frame-by-frame (i.e. one frame per 30ms)"

3. Model 2 and 3 are two nested models, it is redundant having both in the analysis. Compare the two models with respect to a given criterion (such as AIC or BIC) and only include the one with the better score. Probably it will be Model 2 since the third predictor Duration ICU stay is not significant and the Estimate is about zero.
Thank you for the suggestion to compare the models. We reran the analysis and compared all models by using Akaike Information Criterion (AIC). Model 2 does provide a better approximate model compared to Model 3, performing ~ 2x better for both tactile and for nociceptive threshold prediction. Notably, AICc delta between Models 1, 2, and 3 was small (< 2 AIC units), and these three models (but not Model 4) fell within a 87% confidence fit overall.

The model selection AIC data are shown for tactile threshold and nociceptive threshold in the tables below:

(1) Tactile threshold:

Rank	Model	k	AICc	AICc delta	AICc weight	Cumulative AICc weight	Likelihood	Evidence Ratio
1	Model-2: age + GA.hrs	5	232.51	-	0.37	0.37	-110.98	-
2	Model-1: age + surgeries.n	5	232.92	0.41	0.30	0.67	-111.18	1.23
3	Model-3: age + GA.hrs + ICU.days	6	233.84	1.34	0.19	0.87	-110.53	1.95
4	Model 4: age + sex + state	6	234.55	2.04	0.13	1.00	-111.88	2.77

(2) Nociceptive threshold:

Rank	Model	k	AICc	AICc delta	AICc weight	Cumulative AICc weight	Likelihood	Evidence Ratio
1	Model-2: age + GA.hrs	5	167.66	-	0.34	0.34	-78.6	-
2	Model-1: age + surgeries.n	5	167.93	0.06	0.33	0.66	-78.63	1.03
3	Model-3: age + GA.hrs + ICU.days	6	169.04	0.94	0.21	0.87	-77.95	1.6
4	Model 4: age + sex + state	6	169.89	1.92	0.13	1.00	-78.45	2.61

We thank the reviewer for their suggestion and have edited the manuscript to include the results of the AIC analysis accordingly. We have opted to report the statistics for Models 1, 2, and 3 in the main body of the manuscript. The methods section and results sections are updated accordingly.

We have edited the methods to include the following:

> "Models were compared to select the best approximating model based on Akaike Information Criterion (AIC) (ref). AIC considers how well candidate models fit the dataset relative to each of the other models by using likelihood estimation and the number of fitted parameters. Sample-size adjusted AIC (AIC_c) scores were ranked (the smaller the value, the better the model). The relative strength of each candidate model was compared to the top ranked model based on the difference in AIC_c values (Δ_i), Akaike weight, and Evidence Ratio. Criterion for accepting a model were (1) $\Delta_i < 2$ AIC units (indicating the model is as good as the best approximating model); and (2) cumulative Akaike weight < 0.95 (ref). ER provides a measure of how much more likely the best model is than other models. Models 1, 2 and 3 had $\Delta_i < 2$ AIC units, fell within cumulative Akaike weights of 0.87 (indicating 87% confidence that one of the remaining models is the best approximating model), and were considered acceptable for predicting tactile and nociceptive threshold. Model 4 did not satisfy AIC criteria. Model selection was tested using the *AICcmodavg* R package (ref). "

We have edited the results to include the following:

> "Model selection identified Model 2 as the best-fit model; this model included age and cumulative hours of general anaesthesia as fixed effects. Notably, all models had a similar performance given that AICc delta was small (< 2 AIC units). For tactile threshold modelling, Model 2 contained 37% of the cumulative model weight, and the evidence ratio (i.e. how much better Model 2 is compared to other models) for Model 1 was 1.23, and for Model 3 was 1.95. For nociceptive threshold modelling, Model 2 had 34% of the cumulative model weight, and the evidence ratio for Model 1 was 1.03 and for Model 3 was 1.6."

4. In Table 3 Nociceptive threshold there must be a typo, 95% of Duration ICU stay cannot range from 0.01 to 0.01 (I guess the lower CI is -0.01).
Thank you. We cross checked the data output and confirm there was a typo (0.00 instead of 0.01). We have updated the table.

5. In future work it may be of interest to evaluate if similar responses can be evoked with vibration stimuli at difference amplitude and frequency (e.g. delivered with Biothesiometer or similar devices) and/or thermal stimuli.
Thank you for the suggestion. We agree. We currently have IRB approval to pilot a study examining infant cutaneous sensitivity using a range of stimulus modalities including vibration and heat/cool with EMG and video capture. We have edited the Limitations section to include the following:

> "Future research should examine cutaneous sensitivity over a range of modalities including vibration and thermal heat/cool, and in older infants to understand the development of somatosensory processing beyond early infancy."

Minor issues

1. Inpatient (no history of GA exposure), Inpatient (GA-exposed), and Controls (i.e., no previous hospital admission and no-GA). Report in the text of the manuscript the sample size of each group (now it is only in the figure).
Sample sizes for each cohort are provided in Table 1 and in Figure 1. We have edited the Results text to include the sample size of each group.

2. No power calculation the paragraph can be removed
We have deleted the statement on power calculation.
